# Child abuse and neglect experts’ determination of when a child being left home alone constitutes child neglect

**DOI:** 10.1186/s40621-018-0144-0

**Published:** 2018-04-10

**Authors:** Charles A. Jennissen, Erin Evans, Resmiye Oral, Gerene Denning

**Affiliations:** 1 Department of Emergency Medicine, Roy J. and Lucille A. Carver College of Medicine, University of Iowa, Iowa City, IA USA; 2 Roy J. and Lucille A. Carver College of Medicine, University of Iowa, Iowa City, IA USA; 3 Department of Pediatrics, Roy J. and Lucille A. Carver College of Medicine, University of Iowa, Iowa City, IA USA

**Keywords:** Child neglect, Home alone, Children, Parents, Injury, Laws, Legislation, Safety, Child abuse and neglect experts

## Abstract

**Background:**

Only 14 states have laws or guidelines regarding the minimum age a child may be left home alone. These ages range from 6 to 14 years. Our objective was to identify factors that influence child neglect determination by experts with regards to parents leaving children home alone.

**Methods:**

American Academy of Pediatrics Section on Child Abuse and Neglect members (*N* = 523) were surveyed from July–August, 2015. Respondents were asked whether scenarios involving a child of varying age knowingly left home alone for 4 h were neglect in the presence or absence of injury to the child and the legality of the situation. Comparisons were performed using the chi-square test.

**Results:**

One hundred ninety-three members responded (36.9%). In the scenario where there were no relevant laws and the child was uninjured, nearly 100% of the child experts determined this as being child neglect when the child was 6 years old. For 8, 10, 12, and 14 year olds, this was 88, 48, 4, and 1%, respectively. However, a significantly higher percentage of experts considered it child neglect for most ages when there was a law making the scenario illegal as compared when there was no law, and when the child was injured versus when they were not. The only demographic variable that showed a difference in child neglect determination was that females were more likely to consider higher aged children as having been neglected when there were no laws but the child was injured. The vast majority of experts (85%) stated that leaving a child home alone for 4 h should be illegal if the child is < 9 years old, and nearly one-half (44%) said it should be illegal for children < 11 years old.

**Conclusions:**

A number of factors affect how experts view children being left home alone as potential child neglect. Our data suggests that such cases may be evaluated differently due to variations in state laws, even though the risk to the child is the same. These results call for child safety law reform to provide greater uniformity in the evaluation of potential child neglect cases and better protect the safety of children.

## Background

One way in which society enforces expected baseline norms of child rearing and investigates potential child abuse and neglect is through the child welfare and protection system. Mandatory reporters and social workers in governmental agencies, such as the Department of Human Services or Child Protective Services (CPS), are required to report and investigate incidents of child neglect. Essential elements of the child protection system are state and federal laws and guidelines that help establish minimum standards of childcare and identify in many instances what is and is not expected of parents (Children’s Bureau, 2014; Best & Skenazy, 2017). In addition to existing laws and guidelines, the level of gravity that the child protection system assigns to certain parental acts of commission or omission contributes to what is determined as being child abuse and neglect. The actions of this system as a whole help establish societal norms and protect children who are not receiving this minimal standard of care. In this way, the child welfare system has the potential to positively shape the behavior of adults caring for children in society.

Unfortunately, state guidelines and legislation regarding what constitutes child neglect vary widely (Children’s Bureau, 2014; Best & Skenazy, 2017). Particularly striking is the absence of any standardized language defining child neglect in situations where parents provide inadequate supervision, allow their child to engage in activities known to be unsafe, and/or fail to protect their child from recognizable dangers.

Five U.S. states have laws regarding a minimum age below which children may not be legally left home alone, but these ages range from 6 to 14 years old (Best & Skenazy, 2017). Another nine states have guidelines, and the remaining states have no relevant laws or guidelines (Best & Skenazy, 2017). The lack of consistent legislation may hamper the protection of children by failing to define society’s minimal expectations of parents. Moreover, some individuals may argue that the decision to leave a child at home alone should be left up to the parents based on their own judgment of their child’s physical and mental development. However, many parents lack sufficient knowledge of child development to capably determine when their child can safely care for themselves.

A national randomized telephone survey of over 3000 U.S. English and Spanish speaking adults in 2007–2008 were asked to think about a typical child and indicate the minimum age that they could be left alone in their home without an adult (Mack et al., 2012). Overall, the average age survey respondents believed a child could be safely left home alone was 13.0 years old (95% CI = 12.9–13.1). However, respondents who had children ≤10 years of age in the home reported a significantly younger mean minimum age for allowing children to be home alone than adults without children in this age group (12.7 vs. 13.1 years). Regardless, these ages are at the upper end of the minimum ages of current laws and guidelines for those states that have them.

A survey study of child death review (CDR) members from five states assessed their determination as to whether unintentional injury-related deaths were due to child neglect using a series of vignettes (Schnitzer et al., 2011). The study goal was to determine the factors that influenced their decision-making. In the scenarios, two children died in a house fire after playing with a lighter when left home alone because (a) the mother had to go to work and the babysitter had canceled at the last minute, and (b) the mother left for “just a minute” to buy cigarettes. For both situations, nearly four-fifths of the CDR members found that the deaths were definitely neglect-related and nearly 100% felt the situation was at least somewhat neglect-related. The age of the children were not stated in the survey.

No previous studies on children left home alone have focused on the opinions of child abuse and neglect experts. To help address this gap in knowledge and further investigate factors that might influence expert determination of what constitutes child neglect for children left home alone, we surveyed members of the American Academy of Pediatrics Section on Child Abuse and Neglect (SOCAN).

## Methods

### Study design

A survey about child neglect was developed, validated, and distributed to 523 SOCAN members as previously described (Evans et al., 2017). Surveys were distributed through the membership list serve and data collection occurred from July to September of 2015. Members were sent three reminders to complete the survey at 2-week intervals. Demographic variables collected in the survey were sex, age, race/ethnicity, training and certification, and whether the respondent was or had been a parent or child guardian. Geographic variables collected were the state in which the participant worked and the population they primarily served (urban, suburban, rural).

### Child home alone scenario

Survey participants were asked to ignore their own state’s laws and to answer questions based solely on their own judgment of the scenario’s stated situation. They were also instructed to assume that the child was physically and developmentally normal for their age, with no behavioral problems.

The basic child home alone scenario read: “A woman notifies the police that her neighbors left their child home alone. Police respond and find a child in the house, with no adults present. The child had been alone for 4 hours. The parents are aware that they left their child at home alone.” Four additional sets of conditions were then provided: (a) no state laws were violated and the child was not injured; (b) no state laws were violated but “the responding officers found the child with a nearly amputated finger and a sharp knife nearby” (i.e. the child was injured); (c) state laws were violated but there was no injury; (d) state laws were violated and the child was injured. For each set of conditions, the child abuse and neglect experts were instructed to check “yes” or “no” next to each age depending on whether they believed the condition constituted child neglect for a child of that age. The ages were 4, 6, 8, 10, 12, and 14 years old.

### Child home alone laws

The child abuse and neglect experts were also asked to provide a response to the following: “It should be illegal to leave a child at home alone for 4 hours under the age of…”. A dropdown menu allowed the respondent to check a single age from 2 to 18 years old or to select “There should be no laws regarding this.”

### Data analysis

Descriptive (frequencies) and bivariate (chi square) analyses were performed using SPSS (IBM Statistics Package for the Social Sciences, v22). All *p* values were two-tailed with significance defined as *p* < 0.05. Missing data were not included in analyses.

## Results

### Respondent characteristics

A total of 193/523 SOCAN members (37%) completed surveys. The demographics provided for the membership and those of the respondents were not significantly different (Table 1). In addition to the demographics shown in Table 1, the majority of respondents were white (165/188, 86%) and 50 years of age and older (109/192, 57%). The area in which they worked was 39% urban (*n* = 73), 19% suburban (*n* = 35), and 43% rural (*n* = 81). More than eight in ten (157/192, 82%) reported being or having been a parent or legal guardian of a child.

**Table 1:** Demographic comparison of the SOCAN membership and survey participants

	SOCAN Membership	Survey Respondents^a^	*p* value^b^
Region			
Northeast	117 (22%)	32 (18%)	0.49
Midwest	126 (24%)	47 (26%)	
South	156 (30%)	62 (34%)	
West	123 (24%)	42 (23%)	
Sex			
Male	162 (31%)	55 (29%)	0.70
Female	361 (69%)	134 (71%)	
Degree			
MD/DO	496 (95%)	185 (97%)	0.36
CPNP	12 (2%)	4 (2%)	
PA/RN	15 (2%)	2 (1%)	

*Abbreviations:* medical doctor, MD; doctor of osteopathy, DO; certified pediatric nurse practitioner, CPNP; physician’s assistant, PA; registered nurse, RN

^a^Column totals may not equal total number of respondents (*N* = 193) because of missing data

^b^Pearson chi square test

### Scenario results

**Fig. 1:**
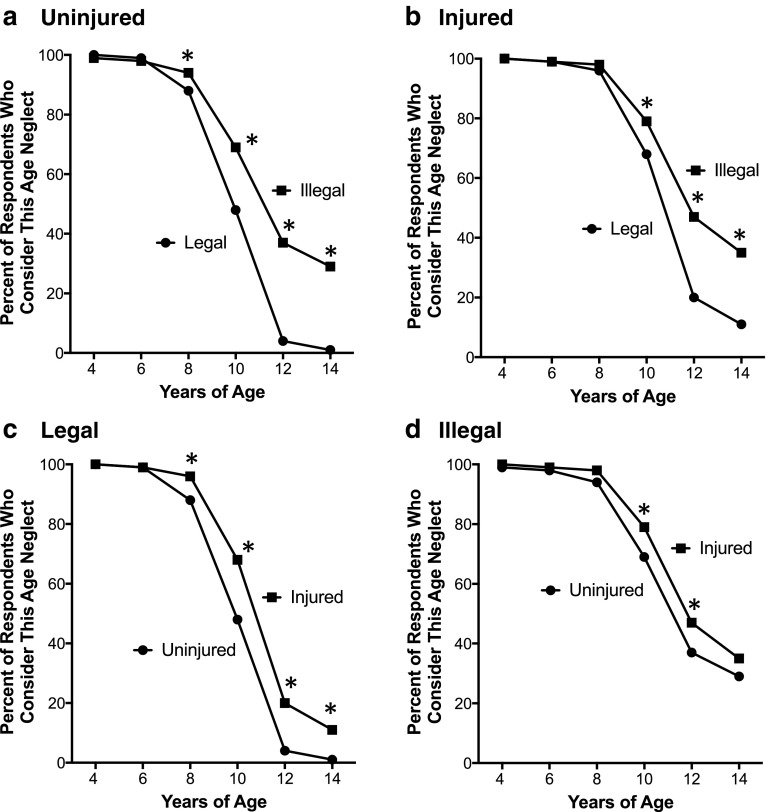
Percent of respondents who considered the indicated age as child abuse, if the child was left home alone for 4 h. The n value for each scenario was 184, unless indicated otherwise. Panels: (**a**) comparison of scenarios involving an uninjured child in the absence (closed circles) and presence (closed squares, *n* = 183) of a “home alone” law covering the selected age; (**b**) comparison of scenarios involving a child that sustained an injury while home alone in the absence (closed circles) and presence (closed squares) of a “home alone” law; (**c**) comparison of scenarios where the child was (closed squares) or was not (closed circles) injured and there was no “home alone” law; (**d**) comparison of scenarios where the child was (closed squares) or was not (closed circles) injured and there was a “home alone” law covering the selected age. Asterisks (*) indicate statistically significant differences (*p* < 0.05) for pairwise comparisons

Figure 1 shows graphs of the proportion of child abuse and neglect experts who included the indicated age as one at which the child being left home alone for 4 h should be determined child neglect under that scenario’s conditions. An asterisk (*) indicates the ages where the two curves are significantly different. The *p* values are provided in Table 2 for age-based comparisons.

**Table 2:** Comparisons between the percentage of respondents who determined the situation as being child neglect for the ages and scenario conditions in the indicated Figure. *P* values were generated using the Pearson chi square test

	*p* value			
	8 years old	10 years old	12 years old	14 years old
Figure 1a^a^	0.030	< 0.0001	< 0.0001	< 0.0001
Figure 1b^b^	0.38	0.032	< 0.0001	< 0.0001
Figure 1c^c^	0.008	< 0.0001	< 0.0001	< 0.0001
Figure 1d^d^	0.099	0.039	0.049	0.27
Figure 2^e^	0.00034	< 0.0001	< 0.0001	< 0.0001

^a^State law versus no state law; the child is uninjured in both cases

^b^State law versus no state law; the child is injured in both cases

^c^Injured versus not injured child; no state law in either case

^d^Injured versus not injured child; state law in both cases

^e^No state law/child uninjured versus state law/child injured

For the scenario condition where there were no relevant “home alone” laws and the child was uninjured (Fig. 1a), nearly 100% of SOCAN members determined that leaving a child home alone for 4 h was child neglect when the child was 6 years of age or younger. For 8, 10, 12, and 14 year olds, this was 88, 48, 4, and 1%, respectively (Fig. 1a). However, having a state law that made it illegal to leave a child of the designated age home alone for 4 h significantly increased the proportion of survey respondents that determined the scenario was child neglect for children ages 8–14 years old. For example, 29% of respondents considered it child neglect to leave a 14 year old home alone when it was illegal, but only 1% considered it child neglect if there was no law.

Even when the child had sustained an injury (Fig. 1b), the additional presence of an applicable law increased the percentage of experts that determined the scenario was child neglect relative to when no law was present. The differences were significant for ages ≥ 10 years old.

The child being injured also significantly increased the proportions of respondents that determined the scenario as being child neglect, and this was true both in the absence (Fig. 1c) and presence (Fig. 1d) of a “home alone” law. In the absence of a law (Fig. 1c), the proportions were significantly higher when the child was ≥ 8 years old if there was an injury versus when there was not. For example, 68% of experts considered it child neglect if a 10 year old had been injured after being left home alone for 4 h even if there was no law, while less than half (48%) determined it to be child neglect if the child was uninjured.

Even when it was illegal to leave a child of the affected age home alone for 4 h (Fig. 1d), the additional presence of an injury to the child increased the percentage of respondents that determined the situation child neglect when 10–12 year olds were involved as compared to when there was no injury to the child.

**Fig. 2:**
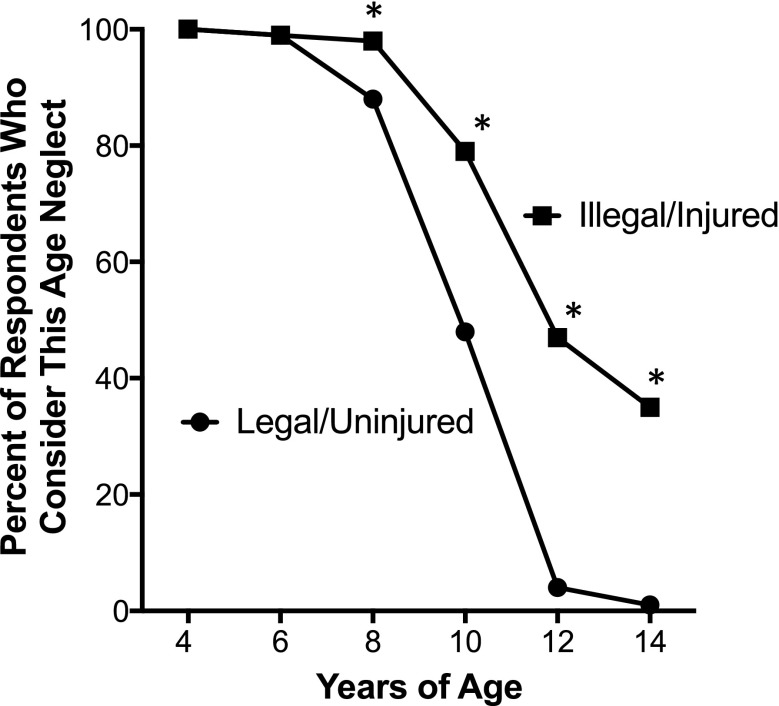
Percent of respondents who considered the indicated age as child abuse, if the child was left home alone for 4 h. The n value for both scenarios was 184. Comparison of a scenario where there was no “home alone” law covering the selected age and the child was not injured (closed circles) and a scenario where the child was injured and there was a relevant “home alone” law (closed squares). Asterisks (*) indicate statistically significant differences (*p* < 0.05) for pairwise comparisons

As expected, the greatest differences were observed when the scenario condition involving an uninjured child and no state law was compared to the condition that included both an applicable state law and an injury to the child (Fig. 2). In that case, almost 80% considered it child neglect to leave a 10-year-old home alone if there was a state law and the child was injured, as compared to less than half (48%) if there was no law and the child was uninjured. Values were 47 and 4% for 12-year-olds, and 35 and 1% for 14-year-olds, respectively.

There were no significant differences in child neglect determination by demographics including sex, race, age, degree/certification, where one worked or having been a parent/legal guardian. The one exception being that in the scenario where there was no state law but the child was injured, a higher proportion of females indicated leaving older children (ages 10–14 years old) home alone should be considered child neglect as compared to males (74% vs. 55%), *p* = 0.043.

### Response to question on child neglect laws

**Fig. 3:**
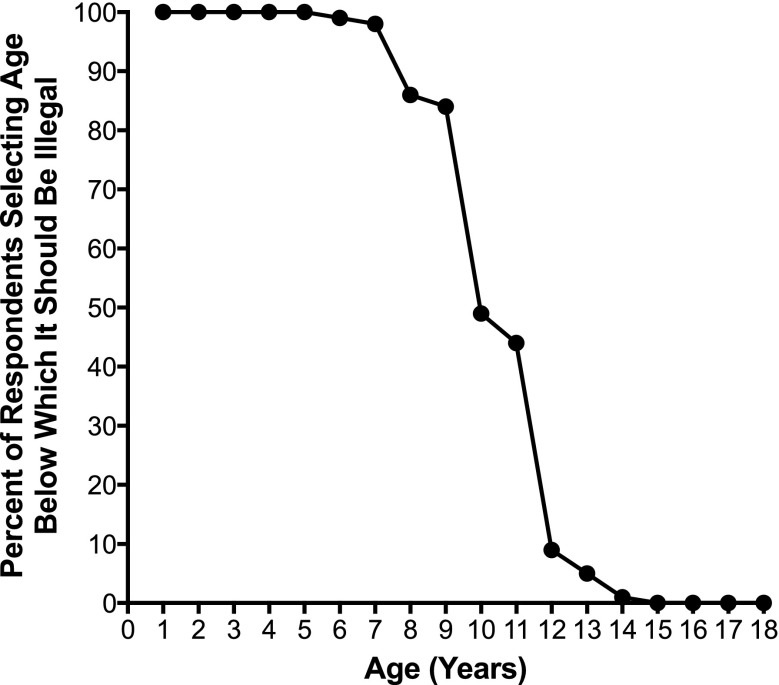
Percent of respondents who selected the indicated age as the age below which it should be illegal to leave a child home alone for 4 h. The n value was 167

Child abuse and neglect experts were asked to select the age below which leaving a child home alone for 4 h should be illegal. Only two of the respondents selected that there should be no law for any age, whereas nearly 100% indicated leaving children less than 7 years of age home alone should be illegal (Fig. 3). In addition, the vast majority (85%) indicated it should be against the law for children less than 9 years of age to be left home alone. However, the percentage recommending a “home alone” law that included 9 year olds dropped to half, and only 5% indicated the law should include 12 year olds and younger. No respondents indicated that laws should make it illegal to leave children 15 years of age and older home alone. There were few differences by demographic variables with the exception that respondents working in urban areas were more likely to choose higher ages (10–14 years) versus lower ages (5–9 years) as needing to be covered by protective child “home alone” laws as compared to those working in suburban (*p* = 0.002) or rural (*p* = 0.006) environments.

## Discussion

Parental supervision is one of the most basic behaviors parents use to protect their children and is an essential element of childhood injury prevention (Mack et al., 2008; Petrass et al., 2009; Saluja et al., 2004; Morrongiello & Schell, 2010; Peterson et al., 1993). Fatality studies have found that over 40% of maltreatment and pediatric injury-related deaths are associated with inadequate supervision (Damashek et al., 2014; Landen et al., 2003). In fact, child neglect is by far the most common reason for referrals to U.S. child protection agencies (Department of Health and Human Services. Administration on Children, Youth and Families, 2017), and in Canada, supervisory neglect was the primary concern in almost one-half of substantiated child neglect and maltreatment cases (Ruiz-Casares et al., 2012).

Despite the importance of parental supervision and its protective effect against injury, there are no generally accepted standards that definitively establish appropriate levels of supervision as children develop. The child neglect literature, including that related to children being left home alone, is highly limited. One study published in 1993 by Peterson et al. surveyed mothers of 8–9 year old children, child protection service workers, and medical personnel (primarily primary care providers) (Peterson et al., 1993). The focus of the study was to assess the participants’ beliefs on what constitutes appropriate parental supervision for children up to 10 years of age in a number of situations with varying levels of risk. They found that respondents across groups generally agreed that children should be left unsupervised for decreasing amounts of time as risk increased, and allowed for increasing amounts of unsupervised time as child age increased. However, the amount of time respondents stated children could be left unsupervised varied significantly especially for the older ages in the study, with some participants being extremely cautious and other outliers with recommendations that might be interpreted by some as reckless. Nonetheless, the study stated that the caregiver in the scenarios was only out of visual and auditory contact, not completely out of the vicinity of the child as is the case with children left home alone.

Leaving a child at home alone means the complete absence of parental supervision. So the question becomes at what age do children reach a level of readiness in cognitive maturity to safely be at home by themselves? Addressing this question requires careful assessment of a number of factors including the child’s behavioral and emotional make-up and their level of executive functioning, e.g. to process information, problem solve, focus attention and control impulses. Many of these frontal lobe functions do not develop until late childhood and may not be fully developed until the mid-20s (Johnson et al., 2009; Tau & Peterson, 2010).

Studies have shown that parents may overestimate their children’s abilities like self-care and impulse control, and knowledge, for example knowing and following through with safety rules (Morrongiello & Schell, 2010; Peterson et al., 1993; Schwebel & Bounds, 2003; Connor & Wesolowski, 2003; Farah et al., 1999; Hardy, 2002; Hardy, 2003; Jackman et al., 2001). Unrealistic parental expectations may be associated with both child neglect and injury (Morrongiello & Schell, 2010; Azar et al., 1984; Azar et al., 2012; Bugental & Happaney, 2004). Also, a child’s decision-making differs substantially from that of adults and can be illustrated by something as routine, yet potentially dangerous, as crossing an intersection on a bicycle (Chihak et al., 2014; Grechkin et al., 2013; Plumert & Kearney, 2014a; Plumert & Kearney, 2014b). Studies have shown that children and adults choose the same size gaps to cross, but children take longer to initiate the crossing causing them to have significantly smaller margins of safety compared to their adult counterparts (Chihak et al., 2014). Thus, children’s delayed cognitive decision-making places them at greater risk than adults when crossing a road. Other factors that should be considered for determining when children can be left home alone would include the length of time the child is to be left alone, whether they will be left alone during a mealtime, how often they will be left alone, whether other children will be allowed to be present, and the child’s ability to respond to an emergency situation (Korioth, 2012).

Child abuse and neglect experts are trained to consider the development and maturity of a child when reviewing a case of potential child neglect. We chose to survey experts in the Child Abuse and Neglect section of the American Academy of Pediatrics. It was our hope that by surveying these experts on what ages and situations constitutes child neglect, we would be able to more accurately define neglect with regards to children being left home alone and provide data that could be used to draft more effective laws and regulations and guide parents in safer child rearing practices.

Our study showed that these child abuse and neglect experts were in fairly strong agreement that leaving children 8 years of age and younger home alone for 4 h was child neglect regardless of existing laws and whether the child was injured. On the other hand, some respondent’s appeared to be influenced by the presence of a “home alone” law, as well as by injury to the child, for ages 8 years and above. This variability is of concern since the risk to the child was not different between the scenario conditions. Our data clearly suggests the need for standardized laws to ensure consistent determinations of child neglect across states. Such laws with consistent enforcement could in turn help prevent deaths and injuries and other negative consequences associated with children being left home alone. As a starting point for discussion, the vast majority of experts (85%) indicated that it should illegal to leave a child at home alone for 4 h if they were less than 9 years of age.

### Limitations

The study was limited to child abuse and neglect experts who were members of AAP SOCAN and may not be generalizable to other populations. It is also possible that some selection bias may have resulted from incomplete membership participation. However, because the demographics provided by the AAP (sex, professional degree and region) were not significantly different for the full membership and the survey respondents, it seems likely that the results reflect the membership as a whole. In our study, chronological age was used as a proxy for the child’s level of development and their acquisition of skills and cognitive maturity to provide self-care and safety. For all of our scenario conditions, we emphasized that the child was physically and developmentally normal for their age, with no behavioral problems. While respondents might differ in how they interpret these criteria, it seems reasonable to assume that overall, respondents will associate increased age and normal development with less need for supervision, more impulse control, and a general increase in skills and abilities that will allow them to be left at home alone with relative safety. In addition, whereas each child expert provided their determination for hypothetical scenarios, it is unclear as to whether their actions in the field evaluating actual cases would reflect their survey responses.

## Conclusions

To our knowledge, this work is the first to assess child abuse and neglect experts’ opinions on child home alone scenarios and so provides a new perspective on this issue. A number of factors, including age of the child, legality of the situation, and presence of injury, affected how experts viewed children being left home alone as potential child neglect. The data suggest that similar cases with equivalent risk may be evaluated differently depending upon the presence or absence of state laws and whether the child was injured or not. Based on these results, it seems reasonable to hypothesize that standardizing child home alone laws across states could help to promote greater uniformity in case evaluations, and thereby more effectively protect children from risks related to being left at home alone.

## Abbreviations

AAP: American Academy of Pediatrics
CDR: Child death review
CPNP: Certified pediatric nurse practitioner
DO: Doctor of osteopathy
MD: Medical doctor
PA: Physician’s assistant
RN: Registered nurse
SOCAN: Section on Child Abuse and Neglect
SPSS: Statistics Package for the Social Sciences
